# Flowering as the Most Highly Sensitive Period of Grapevine (*Vitis vinifera* L. cv Mourvèdre) to the Botryosphaeria Dieback Agents *Neofusicoccum parvum* and *Diplodia seriata* Infection

**DOI:** 10.3390/ijms15069644

**Published:** 2014-05-30

**Authors:** Alessandro Spagnolo, Philippe Larignon, Maryline Magnin-Robert, Agnès Hovasse, Clara Cilindre, Alain Van Dorsselaer, Christophe Clément, Christine Schaeffer-Reiss, Florence Fontaine

**Affiliations:** 1 Université de Reims Champagne-Ardenne, URVVC EA 4707, Laboratoire Stress, Défenses et Reproduction des Plantes, BP 1039, Reims (Cedex 2) 51687, France; E-Mails: alessandro.spagnolo@univ-reims.fr (A.S.); maryline.magnin-robert@univ-reims.fr (M.M.-R.); clara.cilindre@univ-reims.fr (C.Ci.); christophe.clement@univ-reims.fr (C.Cl.); 2 Institut Français de la Vigne et du Vin Pôle Rhône-Méditerranée, France, Domaine de Donadille, Rodilhan 30230, France; E-Mail: Philippe.LARIGNON@vignevin.com; 3 Université de Strasbourg, IPHC, UMR 7178, Laboratoire de Spectrométrie de Masse Bioorganique, Strasbourg 67087, France; E-Mails: ahovasse@unistra.fr (A.H.); alain.vandorsselaer@unistrat.fr (A.V.D.); christine.schaeffer@unistra.fr (C.S.-R.); 4 Université de Reims Champagne-Ardenne, URVVC EA 4707, Laboratoire d’Œnologie et Chimie Appliquée, BP 1039, Reims (Cedex 2) 51687, France

**Keywords:** Botryosphaeria dieback, *Neofusicoccum parvum*, *Diplodia seriata*, plant proteomics, two dimensional gel electrophoresis, defense-related proteins

## Abstract

Botryosphaeria dieback is a fungal grapevine trunk disease that currently represents a threat for viticulture worldwide because of the important economical losses due to reduced yield of affected plants and their premature death. *Neofusicoccum parvum* and *Diplodia seriata* are among the causal agents. Vine green stems were artificially infected with *N. parvum* or *D. seriata* at the onset of three different phenological stages (G stage (separated clusters), flowering and veraison). Highest mean lesion lengths were recorded at flowering. Major proteome changes associated to artificial infections during the three different phenological stages were also reported using two dimensional gel electrophoresis (2D)-based analysis. Twenty (G stage), 15 (flowering) and 13 (veraison) differentially expressed protein spots were subjected to nanoLC-MS/MS and a total of 247, 54 and 25 proteins were respectively identified. At flowering, a weaker response to the infection was likely activated as compared to the other stages, and some defense-related proteins were even down regulated (e.g., superoxide dismutase, major latex-like protein, and pathogenesis related protein 10). Globally, the flowering period seemed to represent the period of highest sensitivity of grapevine to Botryosphaeria dieback agent infection, possibly being related to the high metabolic activity in the inflorescences.

## 1. Introduction

Species in the Botryosphaeriaceae [1,2] have a cosmopolitan distribution and occur as endophytes or pathogens for a wide range of annual and perennial hosts [3]. To date, at least 30 Botryosphaeriaceae species are known to infect grapevine [4,5,6,7,8,9]. Symptoms of Botryosphaeria dieback consist in woody tissues of grey sectorial necrosis with a brown stripe under the bark as well as typical foliar discolorations in white and red cultivars or wilting leaves in some cases [10,11]. *Diplodia seriata* De Not. 1842 and *Neofusicoccum parvum* [1] are among the Botryosphaeria dieback agents more commonly isolated from grapevine-growing regions worldwide ([11] and references therein). Pathogenicity studies indicate that *N. parvum* is among the most virulent Botryosphaeriaceae species to grapevine while *D. seriata* is ranked among those moderately virulent ([7,12,13] and references therein).

Little information is available about the life cycle of Botryosphaeriaceae. The principal sources of inoculum, pycnidia, are located on infected wood, old pruning wounds, and on pruning canes [14]. These fungi are also present at the surface of different organs like canes [15]. The airborne inoculum is present especially during rainfall [16,17] with peak release during the vegetative growth period, especially in France as described by Larignon and Dubos [18] and Kuntzmann *et al.* [19]. Their manner of penetration remains unclear but the most obvious seems to be through pruning wounds in the vineyard [20], notably for *D. seriata* (Ds) and *N. parvum* (Np). In French vineyards, it was shown that *D. seriata* contaminated pruning wounds more often after the bleeding when the mean temperature was above 10 °C and in the presence of rainfall [21]. In these conditions, the susceptibility of pruning wounds was at least 8 weeks. In Italy, wound susceptibility was shown to remain high for up to 4 months after pruning, even in late spring when vines were bleeding [22]. Moreover, fresh wound susceptibility was shown to be greater in spring than in winter. Other wounds caused in vegetative growth period (*i.e*., removal of lateral shoots and desuckering) or by climatic events such as strong wind and hail, could be additional means of fungal penetration inside the vine.

Botryosphaeria dieback is one of the major fungal grapevine trunk diseases which represent a threat for vineyards worldwide due to the decreased production of affected plants and their premature death. Therefore, studying the impact of this disease on grapevine physiology represents a key step towards a better knowledge of symptom development leading to possible control strategies. Phytopathogen infection leads to changes in secondary metabolism based on the induction of a defense programme, as well as to changes in primary metabolism which affect the growth and development of the plant [23]. At the main crop growth stages such as the flowering, the beginning of fruit growth and then their maturity, the physiological status of the plant may be taken into account to elucidate the fine-tuned infection mechanisms. Responsiveness of grapevine infection by some pathogens such as *Botrytis cinerea* at both flowering and berry ripening may originate from a disruption of defense responses and sugar metabolism [24,25].

In this paper, the differential sensitivity of 15-year-old standing vines cv. Mourvèdre to the Botryosphaeria dieback agent infection depending on the phenological stage was assessed by artificial infections with *N. parvum* or *D. seriata* during three vegetative periods (G stage (separated clusters), flowering and veraison). Moreover, in order to gain a better knowledge on the impact of these pathogens on grapevine physiology, major proteome changes in green stems artificially infected were investigated by using a two dimensional gel electrophoresis (2D)-based approach.

## 2. Results and Discussion

### 2.1. Pathogenicity Tests

**Figure 1:**
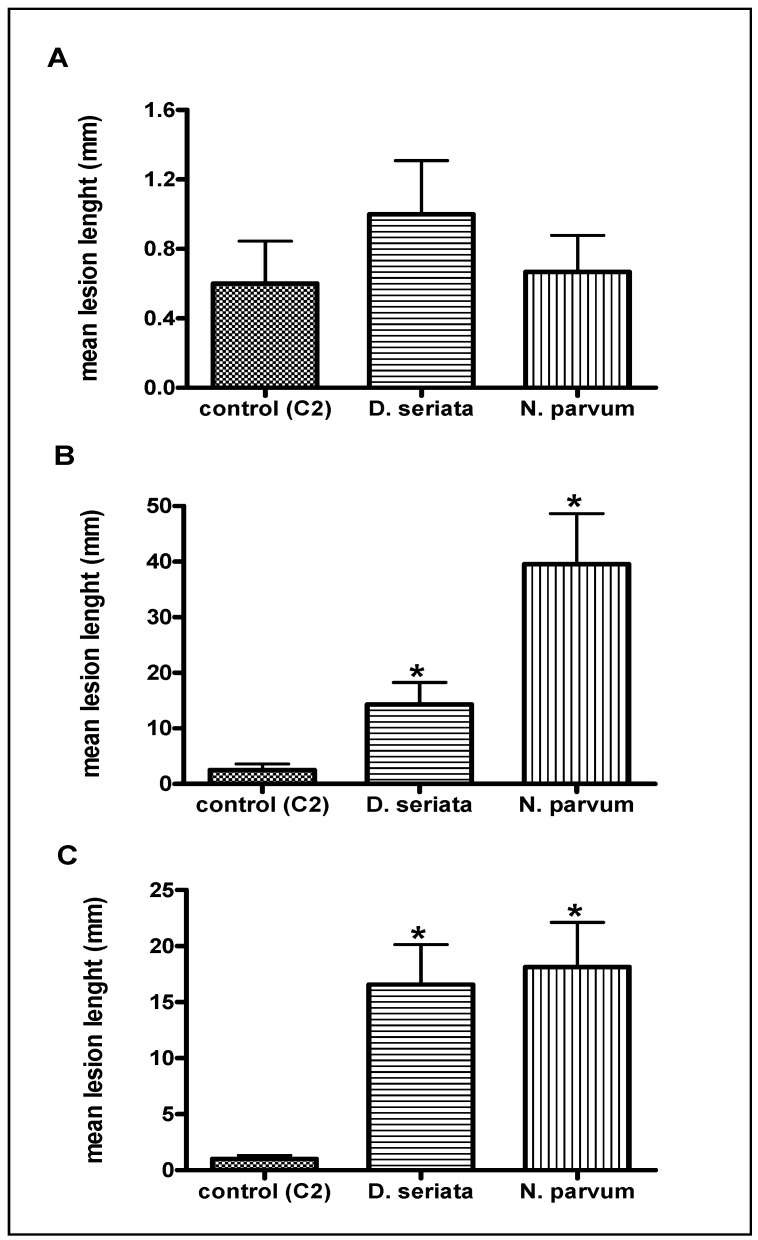
Mean lesion lengths ± SE on green stems after artificial infection with *N. parvum* or *D. seriata* at the onset of the G stage (**A**); flowering (**B**) and veraison (**C**). Control stems were wounded and inoculated with sterile malt agar. Differences among the means were evaluated by the Dunn’s Multiple Comparison Test after that the null hypothesis (equal means) was rejected in the Kruskal–Wallis test, assuming a significance of *p* ≤ 0.05. Statistically significant differences between two conditions are indicated by an asterisk.

The causal association between lesion development and fungal strains was confirmed by the re-isolation from the edge of the lesions. No fungi were isolated from the lesion of control stems. Highest mean lesion length (39.6 ± 9.1 mm) was associated to the Np infection performed at the onset of the flowering (Figure 1). Mean lesion length associated to the Ds infection in the same period was 14.3 ± 3.9 mm while in the case of the control was 2.5 ± 1.1 mm. Lowest mean lesion lengths were registered for the G stage; 0.6 ± 0.2, 1.0 ± 0.3 and 0.7 ± 0.2 mm were measured for the control, Ds and Np treatments, respectively. Intermediate values of lesion length were registered for the control and Np treatments performed at the onset of the veraison, 1.0 ± 0.3 and 18.1 ± 4.0 mm, respectively. Surprisingly, with a size of 16.6 ± 3.6 mm registered at veraison, the necrosis length provoked by Ds was very close to that observed on stem infected with the same fungus at the flowering stage (Figure 1B,C). These results confirm that the degree of the virulence of Np and Ds is different ([7,12,13] and references therein) and probably not based on the same factors. Necrosis development may be influenced by the virulence of the fungal strain in relation to the plant phenological stage. Thus, if the development of lesions is regarded as the expression of the pathogenic potential of these fungi, this result may represent a further indication of the high sensitivity of grapevine to stresses especially during the flowering stage [24]. In this way, several authors hypothesized that high susceptibility of grapevine flowers to biotic stress (necrotrophic fungus *Botrytis cinerea*), may be partially related to their poor ability to carry out the induction of efficient defenses (stilbenic compounds, pathogenesis-related protein) [25,26]. The low responsiveness of flowers to activate the defense responses could be explained by the induction of various mechanisms during flowering period, like fertilization, end of pollen maturation and transition in the whole plant physiology, since the carbohydrate source originating from root and trunk reserves is progressively replaced by photosynthesis in the leaves [24,27,28]. Therefore, the concentrations of sucrose, glucose and fructose, the major sugars of both xylem and phloem saps, are important during the flowering [24]. These carbon sources may represent an attractive nutrient for the fungi. A range of polysaccharide-degrading enzymes and glycosidases were constitutively produced *in vitro* on a simple carbon source like d-glucose by phytopathogenic fungi [29]. Therefore, high production of hydrolytic enzymes induced by high concentration of sugars in the sap, may also explain the highest lesion length observed in stems in response to artificial infection with Ds or Np. Lastly, lesions associated to infections performed at the onset of the G stage did not evolve during the flowering, indicating that the activation of physical and chemical defenses by the plant during the G stage efficiently limited the fungal colonization even during the flowering.

### 2.2. Two-Dimensional Gel Electrophoresis (2D) Analysis

#### 2.2.1. Identification of Differentially Expressed Protein Spots by nanoLC-MS/MS Analysis

**Figure 2:**
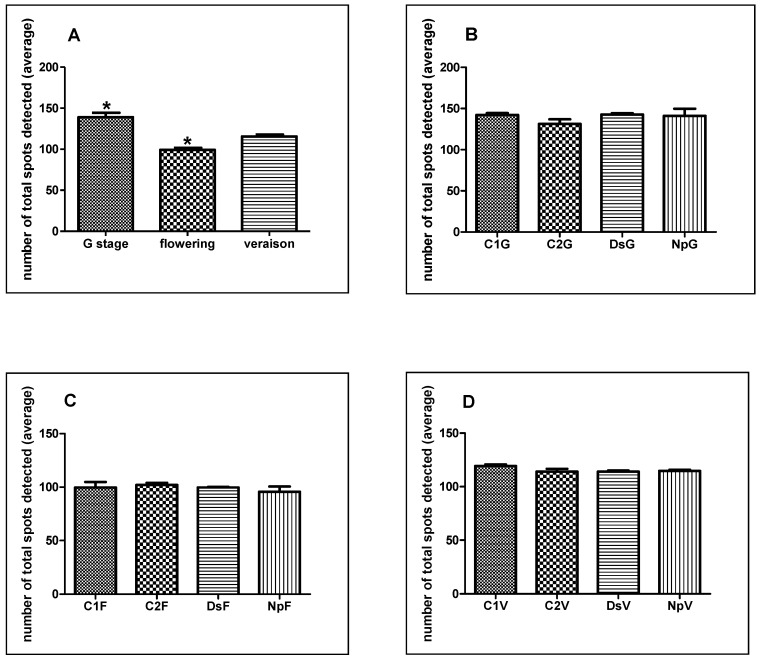
Number of total spots detected on 2D gels from G stage, flowering and veraison including means (mean of three biological replicates) from all the groups in each stage (**A**); Number of total spots detected on 2D gels of each group from G stage (**B**); flowering (**C**) and veraison (**D**). Differences among the means were evaluated by the Dunn’s Multiple Comparison Test after that the null hypothesis (equal means) was rejected in the Kruskal-Wallis test, assuming a significance of *p* ≤ 0.05. Statistically significant differences between two conditions are indicated by an asterisk.

The highest average number of total spots detected in gels concerned the G stage (139) while 99 and 115 spots were recorded for the flowering and the veraison stages, respectively (Figure 2A). The lowest total number of spots detected for the flowering stage could result from a physiological decrease of metabolic activities in the green stem as a consequence of their increasing in the inflorescences. The flowering stage corresponds to an important transition between two distinct and successive phases on sugar physiology during the grapevine cycle. Phase one corresponds to the mobilization of starch from woody organs which supplies the annual organs with carbohydrates during their early growth. Phase two coincides with net leaf photosynthesis which supports both the continuation of annual organs growth and the replenishment of reserves [24].

**Figure 3:**
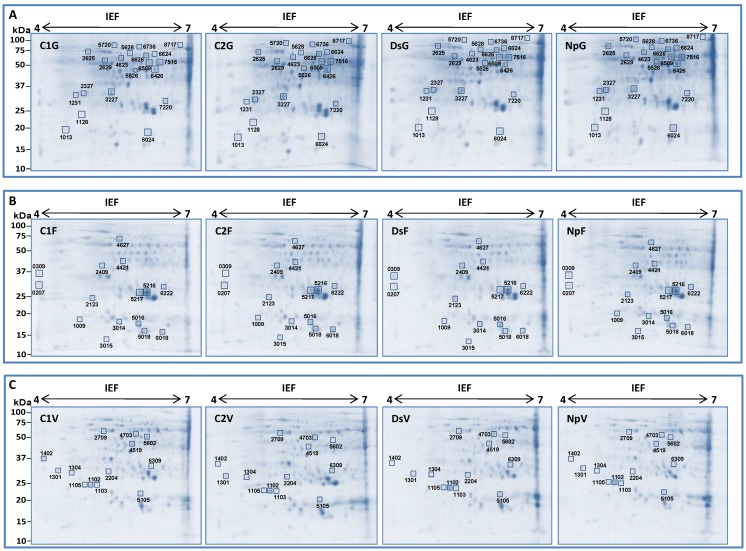
Map of the identified protein spots quantitatively differentially expressed in the green stems of control or infected plants by *Neofusicoccum parvum* (Np) or *Diplodia seriata* (Ds) at G stage (**A**); flowering (**B**) and veraison (**C**). Isoelectric focusing (IEF) was performed on precast dry polyacrylamide 7 cm length gels ReadyStrip IPG (pH 4–7). The relative molecular mass (kDa) was calibrated with standard protein markers (Prestained SDS-PAGE Standards, Bio-Rad, Hercules, CA, USA) after co**-**second dimensional electrophoresis. Only spots detected in at least two biological replicates were chosen for identification (indicated with a square). Spots that were not detected in any gel of a given group are indicated with a circle.

When an in-stage comparison was performed, no statistically significant differences related to the treatment were recorded for any stage (Figure 2B–D). The difference of the number of spots detected was likely related to the stage but not to treatments. Therefore, the number of spots chosen for nanoLC-MS/MS analysis was not related to the average of total spots detected. In fact, we selected 20, 15 and 13 spots for G, flowering and veraison stages, respectively (Figure 3). This could indicate that proteome changes linked to the treatments (C2, DsG, NpG) depend on the phenological stage as they were more pronounced during the G stage. In detail, very few proteins are found to be common to the various stages of development. Only four of the differentially expressed proteins identified in stems were observed in two or three stages. Among them, three were identified in the stem at both G stage and flowering (isoflavone reductase, pathogenesis-related protein 10 (PRP-10) and chaperonin CPN60-2) (Table 1 and Table 2), one identified at G stage and veraison (peroxidase 12-like) and one was identified during the three stages studied (l-ascorbate peroxidase 2, ascoPOX2) (Table 1, Table 2 and Table 3). These results were in accordance with an interesting work on the genome-wide transcriptomic atlas of grapevine, wherein the authors explained that the majority of the genes expressed in berries, tendrils or stems were more common among different organs than at different developmental stages in the same organ [30].

**Figure 4:**
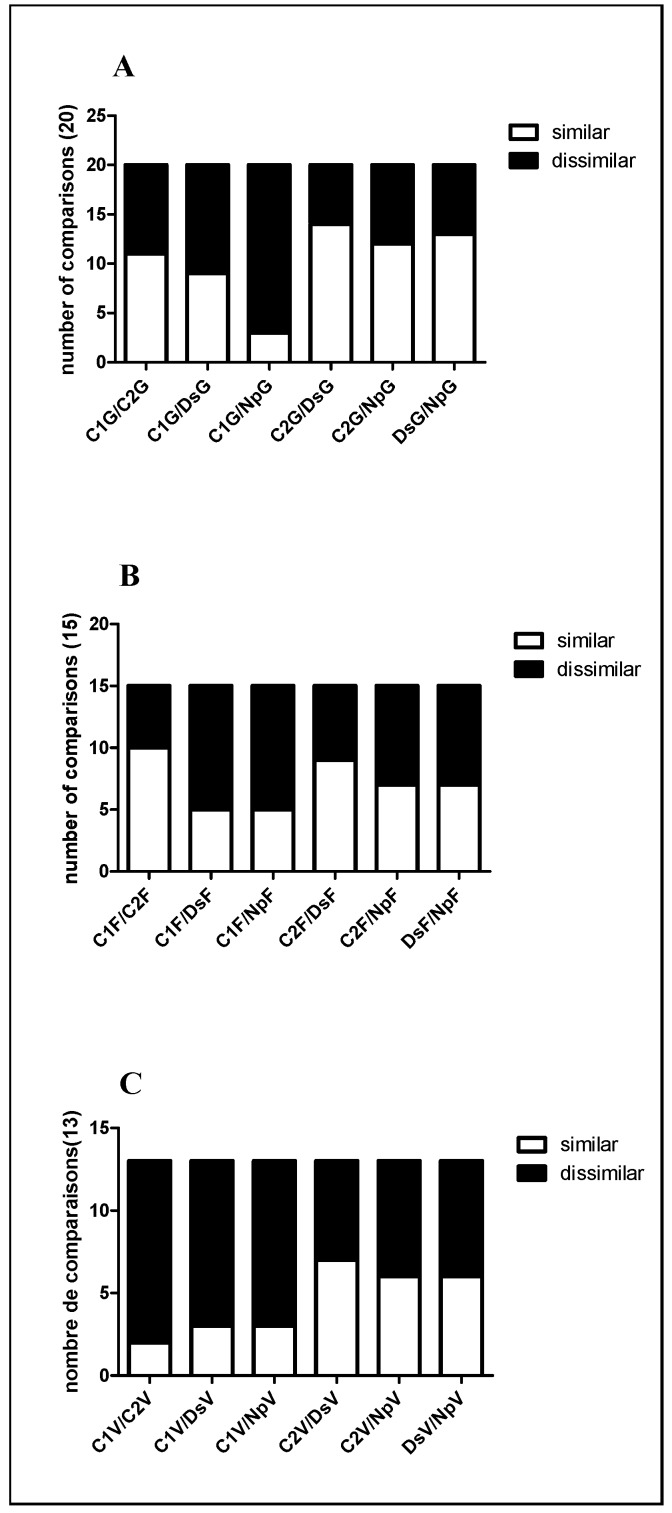
Pairwise comparison for all the possible pairs of group samples in one spot performed for all the identified spots in each stage. G stage (**A**); flowering (**B**), veraison (**C**). The number of spots where their relative expression was considered to be similar (ratio ≤ |2|) or dissimilar (ratio ≥ |2|) is reported.

Protein spot abundance in stems infected with Np or Ds was similar and differences apparently related to the specific inoculum were therefore more pronounced in samples from the G stage (Figure 4). Changes occurred not only in stems infected with Np or Ds but also in C2 (stem wounded but non infected), especially at the veraison stage.

The pairwise comparisons C2/Ds, C2/Np and Ds/Np (for stages G, F and V) showed that specific changes were observed in each treatment but were also common to two or three indicating that changes related to the wounding as well as to the specific inocula occurred at the same time.

#### 2.2.2. Differentially Expressed Proteins among the Treatments

The nanoLC-MS/MS analysis allowed the identification of 247, 54 and 25 differentially expressed total proteins from the G, flowering and veraison stages, respectively (Table S1). A selection of these proteins is listed in Table 1, Table 2 and Table 3.

##### G Stage (Separated Clusters)

No down accumulation was observed in the samples from the G stage (C2G, DsG and NpG) as compared to C1G. Some qualitative differences were detected only in the case of the G stage, the spot s5526 was not detected in C1G. Among the proteins therein identified were a eukaryotic initiation factor, a protein disulfide isomerase, and a peroxidase 12-like. As shown in Table 1, a group of proteins involved in primary amino-acid metabolism was increased by wounding and/or fungal infection only during G stage (s6509, s7615, s5720, s2327). These metabolisms did not seem to be activated by the same treatments at flowering or veraison stages (Table 2 and Table 3). The most notable protein of this group was the γ-amino-butyric acid (GABA) biosynthetic protein glutamate decarboxylase-like (s6509, Table 1), already observed to be accumulated in trunk of grapevine “Chardonnay” affected by Botryosphaeria dieback [31]. While many studies corroborate the link between primary metabolism and plant defense reactions [32,33], the role of primary amino acid metabolism in modulation of defense responses by the host remains scarce. In this sense, glutamate metabolism is known to play an important role in plant amino acid metabolism, orchestrating metabolic functions such as providing both C skeletons and α-amino groups for the biosynthesis of amino acids with key roles in plant defenses: GABA, arginine or proline [34]. Classified in the methionine synthesis pathway, an adenosylhomocysteine isoform 1 (s6509–s7516) was identified in stems and over-accumulated in samples infected by fungi (DsG and NpG). Also associated with the methionine synthesis pathway, *S*-adenosylmethionine (SAM) synthase isoform 2 was abundant in C2G, DsG and NpG (s5526–s6509) (Table 1), as recently reported in green stem and trunk of apoplectic or esca proper-affected grapevine “Chardonnay” [35,36]. SAM synthase leads to the biosynthesis of SAM, which can be metabolised via various pathways, leading to ethylene, phenylpropanoids and polyamines synthesis, important molecules of plant defense responses [37,38]. In this sense, a putative role of the SAM synthase in the intrinsic resistance capability against Ds and Np may be suggested as well as in *Erysiphe necator*- and *Plasmopara viticola*-resistant grapevine cultivar (“Regent”) [39].

Numerous studies highlight that the rate of photosynthesis is reduced locally in response to pathogens [32]. However, in G stage, three proteins involved in photosynthesis were up-regulated in response to fungal infection (Table 1), oxygen-evolving enhancer protein 1 (s1128–s8717), ribulose biphosphate carboxylase (Rubisco) large chain (s7516) and Rubisco large subunit-binding protein subunit alpha (s2629). In grapevine leaves, similar induction of responses related to energy photosynthesis was observed at gene levels, in response to the vascular ascomycete *Eutypa lata* and it appeared to be linked to lack of symptom development on leafy shoots of infected grapevine [40]. At this point, it is reasonable to assume that the rate of photosynthesis could increase to supply the carbon skeletons, energy, and reducing equivalents required to support effective plant defense [33]. Under stress conditions, the respiration process could be enhanced as suggested by increased abundance of glycolysis-related proteins; 2,3-biphosphoglycerate-independent phosphoglycerate mutase (s6624, s5628, s6626) and phosphoglucomutase cytoplasmic (s6624) by wounding, Np and Ds; enolase (s6509) by Np and Ds; and Uridine 5'-diphosphate-glucose dehydrogenase (UDP-Glc DH, s7516, by Ds and Np) from the pentose-phosphate pathway (Table 1). Enolase, an integral enzyme in glycolysis that catalyzes the interconversion of 2-phosphoglycerate to phosphoenolpyruvate, has been found to be responsive in grapevine to phytoplasma infection [41]. Up-regulation of catalyzing enzymes might also increase the production of energy, which is needed in response to Ds and Np infection. Additional studies on UDP-Glc DH indicate that the oxidation of UDP-Glc may have a significant role in increasing the pool of UDP-sugars to supply the demand for increased matrix polysaccharide and cellulose synthesis of structural polysaccharides in plants [42,43]. Moreover, accumulation of GDP-mannose 3,5 epimerase (s6426) and beta-xylosidase/alpha-l-arabinofurasidase (s6626) in stems infected by Np confirmed the activation of cell-wall biosynthesis/modification [44,45,46].

The significant increase in the abundance of proteins related to active oxygen species (AOS), such as ascoPOX2 (s7220), catalase (s7516), and 2-cys-peroxiredoxin (s1128), indicates the induction of an oxidative stress in response to both wounding and fungal infection in stems at G stage (Table 1). In agreement with our observation, most studies have also reported the induction of an antioxidant system (through catalytic activity, proteins expression or transcripts accumulation) on grapevine organs affected by trunk diseases (leaves [40,47,48,49]; green stem [35]; wood [36]). By scavenging the AOS formations, the plant’s antioxidant system protects against toxic oxygen intermediates [50]. Proteins involved in defense responses (s6024) were over-regulated in both NpG and DsG (PRP-10 and a universal stress protein-1). This is supported by the increase of *PR protein 10* gene expression noted in grapevine cell cultures in response to elicitors produced by *Phaeomoniella chlamydospora*, a fungal agent implicated in esca proper [51]. Universal stress proteins are mediated by defense-related hormone ethylene [52] and gene expression is known to be up-regulated in response to fungal infection [53].

Instead, proteins involved in protein synthesis (60 acidic ribosomal protein P0: s1231, and elongation factor 1-beta 1: s1231, s2327) and in signal transduction (14-3-3 protein: s1231, and 14-3-3 protein 7: s2327) were accumulated especially in DsG (Table 1). The 14-3-3 protein was similarly accumulated in brown striped wood of Botryosphaeria dieback grapevine [31]; this protein is known to function as a regulator of a wide range of target proteins in all eukaryotes, and to accumulate in response to abiotic or biotic stresses in plants [54]. The NpG treatment showed the highest number of proteins over-regulated, 22 out of the 63 proteins (Table 1); this suggests that grapevine metabolism was deeply altered by Np infection. In this sense, the up-regulation of various heat shock proteins (mitochondrial heat shock 70 kDa protein—s5628, heat shock cognate 70 kDa protein isoform 2-2625 and stromal 70 kDa heat shock-related protein—s2625) and a chaperonin CPN60-2 (s4623) were only observed in stems infected by Np. HSPs/chaperonins were involved in protein folding, assembly translocation and degradation, therefore playing a pivotal role in protecting plants against various stresses [55,56]. Herein, the post-translation processes may also be involved in stress resistance. Moreover, another HSP cognate 70 kDa (with 96% of identity with HSP cognate 70 kDa, s2625) was recently shown to have its expression and phosphorylation levels upregulated in grapevine leaves infected by phytoplasma. During interaction between phytoplasma and apple tree leaves, HSP cognate 70 kDa was found to be necessary in establishing basal expression levels of several abscisic acid (ABA)-responsive genes, suggesting that this chaperone might also be involved in plant stress hormone ABA signalling events [57].

##### Flowering

Unlike what was observed in the case of green stems infected (Ds or Np) or only wounded (C2) during the G stage, changes that occurred during the flowering consisted of both down- and over-regulation (Table 2). Nearly 50% of the differentially-accumulated proteins were down-regulated during infection (especially with Ds), possibly reflecting the exploitation of cellular resources and/or the suppression of defense responses [58]. A stem-specific protein (SSP) involved in defense and cell rescue, as well as some proteins involved in protein synthesis, glycolysis, photosynthesis, vitamins or nitrogen metabolisms were down regulated in stems infected with Ds or Np. In regard to their low abundances, we can hypothesize a role of these proteins to make a global response insufficient to avoid development of necrosis. For the SSP (s5217), a similar decrease of protein abundance was already observed in the asymptomatic wood of apoplexy-affected grapevine [36]. Only one protein involved in responses to oxidative stresses, a thioredoxin H-type (s3015), was over-regulated in response to wounding (C2F) or fungal infection (DsF and NpF), while the abundance of a superoxide dismutase (SOD, s5018) decreased in the stem infected by Ds. Reduced expression of SOD was also reported in other organs of the vine affected by trunk diseases, green stems [35], leaves [48,49]) and trunk [36]. Similarly to the G stage, PRP-10 (s5016) and HSPs (s3014, s2123) abundance increased in the stem in response to fungal infection, particularly with Ds (Table 2). In accordance with the role of these proteins in plant tolerance against various stresses, the lack of their strong induction in stems infected by Np could explain the longest lesions caused by Np (Figure 1). Moreover, the HSPs accumulated in response to fungal infection are different (18.2 kDa class I HSP isoform 1, 18.2 kDa class I HSP, mitochondrial 23.6 kDa HSP; Table 2) from those accumulated during G stage (70 kDa proteins, Table 1). These results suggest that plant responses to fungal infection may vary significantly with the phenological plant stage. Nevertheless, during the G stage and flowering period, the abundance of isoflavone reductase, a protein involved in secondary metabolism, was increased by two fold in stems infected with Np (Table 1—s5628 and Table 2—s2409). By comparison with the other perturbed metabolisms, the typical secondary metabolism of the vine, represented by the phenylpropanoid pathway, seems to be similarly regulated in response to fungal infection irrespective of the developmental stage (G or F).

Two proteins of the primary metabolism, cytosolic triosephosphate isomerase (TIP, s6222) and pyruvate dehydrogenase (s2409), were among those over-accumulated mainly in NpF. The TPI allows the reversible isomerization between d-glyceraldehyde-3-phosphate (G3P) and dihydroxyacetone phosphate. Note that the G3P is used in the glycolytic pathway to ultimately give pyruvate. At the end of glycolysis, the generated pyruvate will be subsequently decarboxylated and will react with coenzyme A via the action of the pyruvate dehydrogenase, to give acetyl coenzyme A, the entry point of the Krebs cycle. Accumulation of the proteins associated with glycolysis and the Krebs cycle may support cellular energy requirements for plant defense reactions in response to Np infection [32,33]. Despite their accumulation of proteins, significant necrosis is measured on the stems inoculated with Np which suggests no effect of these proteins in limiting necrosis development.

##### Veraison

Similarly to the other two stages, HSP was over-accumulated during veraison. In addition, a chloroplastic small HSP (s1102–s1103–s1105) was abundant in stems C2V, DsV and NpV (Table 3). In response to fungal infection, a SAM synthase 5 (s5105), a 22.0 kDa HSP (s5105) and a ascoPOX2 (s6309) protein were over-accumulated in DsV and NpV. Considering the expression of these three proteins, it is obvious that their change in the stem has no effective influence in limiting necrosis development. Still, a glutathione *S*-transferase F9 (GSTF9, s2204) was among those over-regulated in both C2V and DsV but not in NpV, while a peroxidase 12-like (s4703) was over-accumulated solely in DsV. Nevertheless, the lesion lengths observed on green stems infected by Ds or Np are very similar and suggest that the accumulation of both proteins does not stop necrosis development. For the GST, various studies proposed that GST system is not related to the plant tolerance against pathogenic fungi [36,47]. In this sense, Spagnolo *et al.* [31] observed no relationship between the up-/down-regulation of GSTF9 and the greater or lesser susceptibility of the grapevine cultivars against Botryosphaeria dieback. Two proteins involved in protein synthesis (s1301, putative transcription factor and nascent polypeptide-associated complex subunit alpha-like) were over- and down-accumulated in C2V and NpV respectively (Table 3). This contrasting response suggests an alteration of the proteins synthesis under infection condition.

**Table 1:** Identified proteins differentially expressed during the G stage.

Spot ^a^	Ratio to C1G ^b^			Matched Protein ^c^	Accession Number ^d^	Cov. % ^e^	*M*_W_^f^	Functional Category
	C2G	DsG	NpG					
5526	*	*	*	*S*-adenosylmethionine synthase 2	gi|223635284	40	24.70	Defense and cell rescue
5526	*	*	*	eukaryotic initiation factor 4A-2 (*Vitis vinifera*)	gi|225442221	46	53.30	Protein synthesis
5526	*	*	*	protein disulfide isomerase-like 2-3 (*Vitis vinifera*)	gi|225447176	46	32.80	Protein processing
5526	*	*	*	peroxidase 12-like (*Vitis vinifera*)	gi|359493149	39	33.10	Defense and cell rescue
1013	9.0	4.6	9.5	actin-depolymerizing factor 2-like isoform 1 (*Vitis vinifera*)	gi|225435040	16	13.70	Cytoskeleton
1128	3.0	7.6	8.3	2-Cys peroxiredoxin (*Vitis vinifera*)	gi|147789752	30	52.70	Defense and cell rescue
1128	3.0	7.6	8.3	oxygen-evolving enhancer protein 1, chloroplastic (*Vitis vinifera*)	gi|147791852	33	20.50	Photosynthesis
1231	4.1	12.3	4.5	60S acidic ribosomal protein P0 (*Vitis vinifera*)	gi|147843260	34	12.50	Protein synthesis
1231	4.1	12.3	4.5	elongation factor 1-beta 1 (*Vitis vinifera*)	gi|29608391	27	38.30	Protein synthesis
1231	4.1	12.3	4.5	14-3-3 protein (*Vitis vinifera*)	gi|359492889	29	66.20	Signal transduction
6624	2.4	2.6	4.5	phosphoglucomutase, cytoplasmic (*Vitis vinifera*)	gi|225424316	63	26.60	Glycolysis/Gluconeogenesis
6624	2.4	2.6	4.5	succinate dehydrogenase (ubiquinone) flavoprotein subunit 1, mitochondrial isoform 1 (*Vitis vinifera*)	gi|225430776	73	38.80	Citrate cycle
6624	2.4	2.6	4.5	2,3-bisphosphoglycerate-independent phosphoglycerate mutase isoform 1 (*Vitis vinifera*)	gi|225439064	61	24.50	Glycolysis/Gluconeogenesis
6736	3.2	3.3	3.4	eukaryotic initiation factor 4A-2 (*Vitis vinifera*)	gi|225442221	46	22.80	Protein synthesis
6736	3.2	3.3	3.4	phospholipase D alpha 1 (*Vitis vinifera*)	gi|225442981	92	41.90	Glycan metabolism
6736	3.2	3.3	3.4	ATP-dependent Clp protease ATP-binding subunit clpA homolog CD4A, chloroplastic-like (*Vitis vinifera*)	gi|225456471	102	46.50	Cell growth and death
8717	3.0	2.0	2.8	oxygen-evolving enhancer protein 1, chloroplastic (*Vitis vinifera*)	gi|147791852	33	36.60	Photosynthesis
8717	3.0	2.0	2.8	elongation factor 2-like isoform 1 (*Vitis vinifera*)	gi|225462164	93	44.70	Protein synthesis
6509	1.1	2.1	2.5	*S*-adenosylmethionine synthase 2	gi|223635284	43	44.00	Defense and cell rescue
6509	1.1	2.1	2.5	UDP-glucose 6-dehydrogenase-like isoform 1 (*Vitis vinifera*)	gi|225423507	52	23.70	Pentose phosphate cycle
6509	1.1	2.1	2.5	adenosylhomocysteinase isoform 1 (*Vitis vinifera*)	gi|225433506	53	30.50	Amino acid metabolism
6509	1.1	2.1	2.5	enolase 1 (*Vitis vinifera*)	gi|225441000	47	56.80	Glycolysis/Gluconeogenesis
6509	1.1	2.1	2.5	glutamate decarboxylase-like (*Vitis vinifera*)	gi|225462892	57	25.30	Amino acid metabolism
6024	1.3	3.0	3.5	pathogenesis-related protein 10 (Vitis hybrid cultivar)	gi|163914213	17	49.40	Defense and cell rescue
6024	1.3	3.0	3.5	universal stress protein A-like protein isoform 1 (*Vitis vinifera*)	gi|225431940	18	57.60	Defense and cell rescue
6024	1.3	3.0	3.5	eukaryotic translation initiation factor 5A (*Vitis vinifera*)	gi|225468027	17	28.10	Protein synthesis
7516	1.2	2.0	2.4	Ribulose bisphosphate carboxylase large chain (*Vitis vinifera*)	gi|134034997	6	40.00	photosynthesis
7516	1.2	2.0	2.4	catalase (*Vitis vinifera*)	gi|19070130	56	12.20	Glyoxylate and dicarboxylate metabolism
7516	1.2	2.0	2.4	ATPase subunit 1 (*Vitis vinifera*)	gi|224365668	55	19.30	Metabolism and energy
7516	1.2	2.0	2.4	UDP-glucose 6-dehydrogenase-like isoform 1 (*Vitis vinifera*)	gi|225423507	52	24.20	Pentose phosphate cycle
7516	1.2	2.0	2.4	adenosylhomocysteinase isoform 1 (*Vitis vinifera*)	gi|225433506	53	38.10	Amino acid metabolism
7516	1.2	2.0	2.4	enolase 1 (*Vitis vinifera*)	gi|225441000	47	40.50	Glycolysis/Gluconeogenesis
5720	2.6	1.7	2.1	phospholipase D alpha 1 (*Vitis vinifera*)	gi|225442981	92	25.50	Glycan metabolism
5720	2.6	1.7	2.1	aminopeptidase N-like (*Vitis vinifera*)	gi|359474189	101	50.60	Amino acid metabolism
3227	2.0	0.7	0.8	oxygen-evolving enhancer protein 1, chloroplastic (*Vitis vinifera*)	gi|147791852	33	57.70	Photosynthesis
3227	2.0	0.7	0.8	isoflavone reductase homolog P3 (*Vitis vinifera*)	gi|225458243	33	34.40	Secondary metabolism
3227	2.0	0.7	0.8	putative fructokinase-5-like (*Vitis vinifera*)	gi|225459906	34	23.20	Carbohydrate metabolism
2327	1.3	3.4	1.6	ran-binding protein 1 homolog c (*Vitis vinifera*)	gi|225439378	24	37.60	Defense and cell rescue
2327	1.3	3.4	1.6	14-3-3 protein 7 (*Vitis vinifera*)	gi|225459292	28	66.30	Signal transduction
2327	1.3	3.4	1.6	elongation factor 1-beta 1 (*Vitis vinifera*)	gi|296083911	27	55.30	photosynthesis
2327	1.3	3.4	1.6	aspartic proteinase isoform 2 (*Vitis vinifera*)	gi|302144105	46	23.50	Amino acid metabolism
7220	1.1	1.6	9.4	triosephosphate isomerase, cytosolic (*Vitis vinifera*)	gi|147784332	27	19.30	Glycolysis/Gluconeogenesis
7220	1.1	1.6	9.4	l-ascorbate peroxidase 2, cytosolic (*Vitis vinifera*)	gi|225435177	27	64.80	Carbohydrate metabolism
7220	1.1	1.6	9.4	3-oxoacyl-(acyl-carrier-protein) reductase 1, chloroplastic (*Vitis vinifera*)	gi|225456248	34	24.80	Fatty acid metabolism
6426	1.3	1.3	2.4	eukaryotic initiation factor 4A-3-like (*Vitis vinifera*)	gi|147785805	44	42.20	Protein synthesis
6426	1.3	1.3	2.4	GDP-mannose 3,5-epimerase 1 isoform 1 (*Vitis vinifera*)	gi|147794688	42	38.30	Carbohydrate metabolism
6426	1.3	1.3	2.4	I-adenosylmethionine synthase 2	gi|223635284	43	83.00	Defense and cell rescue
6426	1.3	1.3	2.4	alcohol dehydrogenase 1 (*Vitis vinifera*)	gi|225431505	41	18.40	Glycolysis/Gluconeogenesis
6426	1.3	1.3	2.4	isocitrate dehydrogenase (NADP) (*Vitis vinifera*)	gi|225466253	42	50.00	Citrate cycle
5628	1.0	1.1	2.3	heat shock 70 kDa protein, mitochondrial-like (*Vitis vinifera*)	gi|225429228	72	41.70	Protein destination
5628	1.0	1.1	2.3	2,3-bisphosphoglycerate-independent phosphoglycerate mutase isoform 1 (*Vitis vinifera*)	gi|225439064	61	22.20	Glycolysis/Gluconeogenesis
5628	1.0	1.1	2.3	isoflavone reductase homolog P3 (*Vitis vinifera*)	gi|225458243	33	14.00	Secondary metabolism
2625	1.3	1.5	2.1	luminal-binding protein 5-like (*Vitis vinifera*)	gi|359490716	73	42.40	
2625	1.3	1.5	2.1	heat shock cognate 70 kDa protein isoform 2 (*Vitis vinifera*)	gi|359486799	75	38.60	Protein destination
2625	1.3	1.5	2.1	stromal 70 kDa heat shock-related protein, chloroplastic-like (*Vitis vinifera*)	gi|225456004	75	53.50	Protein destination
2629	1.2	0.9	2.0	protein disulfide-isomerase (*Vitis vinifera*)	gi|225459587	55	51.50	Protein processing
2629	1.2	0.9	2.0	ruBisCO large subunit-binding protein subunit alpha, chloroplastic-like (*Vitis vinifera*)	gi|359479362	61	67.60	photosynthesis
6626	1.1	1.3	2.0	succinate dehydrogenase (ubiquinone) flavoprotein subunit 1, mitochondrial isoform 1 (*Vitis vinifera*)	gi|225430776	73	12.10	Citrate cycle
6626	1.1	1.3	2.0	2,3-bisphosphoglycerate-independent phosphoglycerate mutase isoform 1 (*Vitis vinifera*)	gi|225439064	61	64.90	Glycolysis/Gluconeogenesis
6626	1.1	1.3	2.0	beta-xylosidase/alpha-l-arabinofuranosidase 2-like (*Vitis vinifera*)	gi|297745522	80	16.60	Carbohydrate metabolism
4623	0.7	0.6	2.0	d-3-phosphoglycerate dehydrogenase, chloroplastic-like (*Vitis vinifera*)	gi|225428898	62	10.60	Glycolysis/Gluconeogenesis
4623	0.7	0.6	2.0	chaperonin CPN60-2, mitochondrial isoform 1 (*Vitis vinifera*)	gi|225433375	61	68.70	Protein destination
4623	0.7	0.6	2.0	stromal 70 kDa heat shock-related protein, chloroplastic-like (*Vitis vinifera*)	gi|225456004	75	20.70	Protein destination

^a^ spot code as reported in Figure 3; ^b^ ratio of spot expression values (relative OD × area%) in C2, Ds and Np stems to the related control (C1G, C1F or C1V). Values indicating over or down expression (ratio ≥ |2|) are highlighted in yellow, respectively. Values were replaced by an asterisk when the spot was not detected in the control; ^c^ protein identified via the MASCOT and OMSSA search engines against in house made database from NCBInr database; ^d^ accession No. of the matched protein as reported in the NCBI database; ^e^ percentage of the protein sequence covered by the matching peptides; ^f^ molecular mass (kDa).

**Table 2:** Identified proteins differentially expressed during flowering.

Spot ^a^		Ratio to C1F ^b^						Matched Protein ^c^		Accession Number ^d^		Cov. % ^e^		*M*_W_^f^		Functional Category	
		C2F		DsF		NpF											
3015		4.56		4.69		2.95		thioredoxin H-type isoform 1 (*Vitis vinifera*)		gi|225458147		62		12.80		Protein folding	
3014		2.80		26.5		1.78		18.2 kDa class I heat shock protein isoform 1 (*Vitis vinifera*)		gi|225449302		46		17.02		Protein destination	
2123		0.83		3.52		1.25		23.6 kDa heat shock protein, mitochondrial isoform 1(*Vitis vinifera*)		gi|225466111		42		23.74		Protein destination	
1009		0.58		0.96		3.47		18.2 kDa class I heat shock protein isoform 1 (*Vitis vinifera*)		gi|225449302		13		17.02		Protein destination	
6222		1.04		0.61		3.05		l-ascorbate peroxidase 2, cytosolic (*Vitis vinifera*)		gi|225435177		65		27.56		Carbohydrate metabolism	
6222		1.04		0.61		3.05		triosephosphate isomerase, cytosolic (*Vitis vinifera*)		gi|225449541		53		21.13		Glycolysis/Gluconeogenesis	
2409		0.70		1.37		2.10		l-galactose dehydrogenase (*Vitis vinifera*)		gi|146432259		47		34.64		Carbohydrate metabolism	
2409		0.70		1.37		2.10		pyruvate dehydrogenase E1 component subunit beta, mitochondrial-like isoform 1 (*Vitis vinifera*)		gi|225425166		42		39.49		Glycolysis/Gluconeogenesis	
2409		0.70		1.37		2.10		PREDICTED: fructokinase-2 (*Vitis vinifera*)		gi|225433918		53		35.20		Carbohydrate metabolism	
2409		0.70		1.37		2.10		isoflavone reductase homolog P3 (*Vitis vinifera*)		gi|225458243		36		33.81		Secondary metabolism	
5016		1.08		3.31		0.46		18.2 kDa class I heat shock protein (*Vitis vinifera*)		gi|225449250		49		18.15		Protein destination	
5016		1.08		3.31		0.46		pathogenesis-related protein 10 (Vitis hybrid cultivar)		gi|163914213		14		17.11		Defense and cell rescue	
4627		0.43		1.08		1.38		MLP-like protein 34 (*Vitis vinifera*)		gi|225424277		47		17.08		Defense and cell rescue	
4627		0.43		1.08		1.38		chaperonin CPN60-2, mitochondrial isoform 1 (*Vitis vinifera*)		gi|225433375		45		61.37		Protein destination	
4627		0.43		1.08		1.38		ruBisCO large subunit-binding protein subunit beta, chloroplastic (*Vitis vinifera*)		gi|225435794		42		64.61		Photosynthesis	
6018		1.90		0.24		1.12		ubiquitin-conjugating enzyme E2 35 isoform 1 (*Vitis vinifera*)		gi|225461646		51		17.22		Protein degradation	
6018		1.90		0.24		1.12		MLP-like protein 34 (*Vitis vinifera*)		gi|225424277		72		17.08		Defense and cell rescue	
6018		1.90		0.24		1.12		chaperonin CPN60-2, mitochondrial isoform 1 (*Vitis vinifera*)		gi|225433375		27		61.37		Protein destination	
6018		1.90		0.24		1.12		ubiquitin-conjugating enzyme E2 36 isoform 1 (*Vitis vinifera*)		gi|225446595		38		17.22		Protein degradation	
5018		1.65		0.17		0.65		MLP-like protein 34 (*Vitis vinifera*)		gi|225424277		76		17.08		Defense and cell rescue	
5018		1.65		0.17		0.65		ubiquitin-conjugating enzyme E2 36 isoform 1 (*Vitis vinifera*)		gi|225446595		38		17.22		Protein degradation	
5018		1.65		0.17		0.65		glycine-rich RNA-binding protein GRP1A-like (*Vitis vinifera*)		gi|359475330		17		16.33		Cell growth and death	
5018		1.65		0.17		0.65		superoxide dismutase (Cu-Zn) isoform 2 (*Vitis vinifera*)		gi|225451120		29		15.28		Defense and cell rescue	
5216		0.73		0.50		0.42		putative transcription factor (*Vitis vinifera*)		gi|14582465		31		16.70		Protein synthesis	
5216		0.73		0.50		0.42		ferritin-3, chloroplastic (*Vitis vinifera*)		gi|147784301		18		25.37		Metabolism of cofactors and vitamins	
5216		0.73		0.50		0.42		triosephosphate isomerase, chloroplastic-like isoform 1 (*Vitis vinifera*)		gi|225427917		59		34.67		Glycolysis/Gluconeogenesis	
4421		0.52		0.47		0.33		glutelin type-A 1 (*Vitis vinifera*)		gi|225435090		44		38.33		-	
4421		0.52		0.47		0.33		glutamine synthetase nodule isozyme isoform 1 (*Vitis vinifera*)		gi|225451235		19		34.37		Nitrogen metabolism	
5217		0.61		0.32		0.19		stem-specific protein TSJT1 (*Vitis vinifera*)		gi|225432548		46		25.25		Defense and cell rescue	
0207		0.20		0.10		0.24		putative ripening-related protein (*Vitis vinifera*)		gi|7406667		23		15.39		-	
0309		0.35		0.24		0.16		uncharacterized protein LOC100232885 (*Vitis vinifera*)		gi|225447003		78		18.41		-	

^a^ spot code as reported in Figure 3; ^b^ ratio of spot expression values (relative OD × area%) in C2, Ds and Np stems to the related control (C1G, C1F or C1V). Values indicating over or down expression (ratio ≥ |2|) are highlighted in yellow or grey, respectively. Values were replaced by an asterisk when the spot was not detected in the control; ^c^ protein identified via the MASCOT and OMSSA search engines against in house made database from NCBInr database; ^d^ accession No. of the matched protein as reported in the NCBI database; ^e^ percentage of the protein sequence covered by the matching peptides; ^f^ molecular mass (kDa).

**Table 3:** Identified proteins differentially expressed during the veraison.

Spot ^a^	Ratio to C1V ^b^			Matched Protein ^c^	Accession Number ^d^	Cov. % ^e^	*M*_W_^f^	Functional Category
	C2V	DsV	NpV					
1102	5.50	4.35	3.26	small heat shock protein, chloroplastic (*Vitis vinifera*)	gi|225455238	55	25.03	Protein destination
1103	12.93	8.10	2.86	small heat shock protein, chloroplastic (*Vitis vinifera*)	gi|225455238	48	25.03	Protein destination
1105	33.75	5.75	18.75	small heat shock protein, chloroplastic (*Vitis vinifera*)	gi|225455238	54	25.03	Protein destination
5105	1.98	3.02	2.16	*S*-adenosylmethionine synthase 5	gi|223635289	10	42.79	Defense and cell rescue
5105	1.98	3.02	2.16	22.0 kDa heat shock protein (*Vitis vinifera*)	gi|225459900	46	21.12	Protein destination
6309	1.95	2.16	2.40	l-ascorbate peroxidase 2, cytosolic (*Vitis vinifera*)	gi|225435177	61	27.56	Other carboyhydrate metabolism
2204	4.13	2.20	1.58	uncharacterized protein LOC100254632 (*Vitis vinifera*)	gi|225441008	34	16.79	
2204	4.13	2.20	1.58	glutathione S-transferase F9 (*Vitis vinifera*)	gi|225446791	29	24.91	Defense and celle rescue
2204	4.13	2.20	1.58	uridylate kinase isoform 1 (*Vitis vinifera*)	gi|225454048	20	23.53	Nucleotide metabolism
4703	1.31	2.91	0.90	peroxidase 12-like (*Vitis vinifera*)	gi|359493149	64	39.18	Defense and cell rescue
1301	2.15	1.29	0.29	putative transcription factor (*Vitis vinifera*)	gi|14582465	14	16.70	Protein synthesis
1301	2.15	1.29	0.29	nascent polypeptide-associated complex subunit alpha-like (*Vitis vinifera*)	gi|225470846	42	22.03	Protein synthesis
2709	0.00	1.34	0.20	tubulin alpha-2 chain (*Vitis vinifera*)	gi|225458970	57	49.59	Cytoskleton
4519	0.20	0.70	0.50	naringenin,2-oxoglutarate 3-dioxygenase (*Vitis vinifera*)	gi|225431140	65	40.81	Secondary metabolism
4519	0.20	0.70	0.50	caffeic acid 3- *O*-methyltransferase 1-like isoform 1 (*Vitis vinifera*)	gi|359490763	53	39.50	Secondary metabolism
1304	0.44	0.07	0.48	proteasome subunit alpha type-5 isoform 1 (*Vitis vinifera*)	gi|225441985	43	25.98	Protein degradation
1402	0.12	0.07	0.27	uncharacterized protein LOC100232885 (*Vitis vinifera*)	gi|225447003	83	18.41	
5602	0.03	0.24	0.16	*S*-adenosylmethionine synthase 5	gi|223635289	75	42.79	Defense and celle rescue

^a^ spot code as reported in Figure 3; ^b^ ratio of spot expression values (relative OD × area%) in C2, Ds and Np stems to the related control (C1G, C1F or C1V). Values indicating over or down expression (ratio ≥ |2|) are highlighted in yellow or grey, respectively. Values were replaced by an asterisk when the spot was not detected in the control; ^c^ protein identified via the MASCOT and OMSSA search engines against in house made database from NCBInr database; ^d^ accession No. of the matched protein as reported in the NCBI database; ^e^ percentage of the protein sequence covered by the matching peptides; ^f^ molecular mass (kDa).

**Figure 5:**
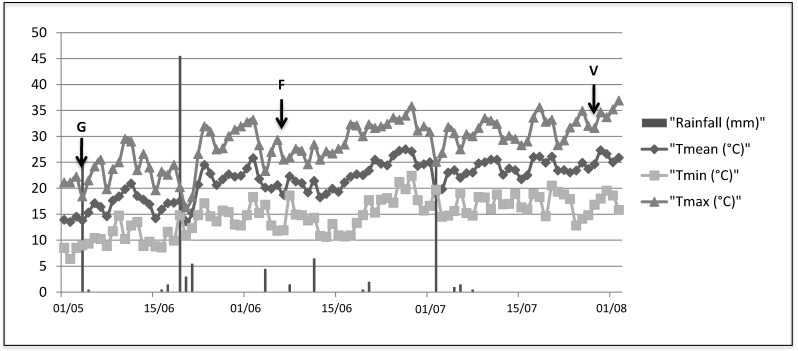
The climate trend [minimum, maximum, mean daily temperatures and rainfall (mm)] recorded in the experimental site (Table 1, Table 2 and Table 3).

## 3. Experimental Section

### 3.1. Plant Material, Fungal Strains and Pathogenicity Tests

A 15-year-old vineyard cultivar Mourvèdre/3309 located at Rodilhan (Costières de Nîmes, France) and owned by the *Lycée* agricole Marie-Durand of Rodilhan was the experimental site. Green stems at the onset of the phenological stage G, flowering and veraison were selected for the study and a total of four treatments were planned: C1, stem non inoculated and non-wounded; C2, stem wounded and inoculated with sterile malt agar plug; Ds, stem inoculated with the *D. seriata* strain (strain Bo98-1 isolated from symptomatic vines in Pyrénées-Orientales vineyards, France) and Np, stem inoculated with the *N. parvum* strain (strain Np SV isolated from symptomatic vines in Bouches-du-Rhône, France) (Table 4). Fungal strains were cultivated on 1.5% malt extract agar at 24 °C in the dark. Stems were inoculated at the onset of G stage (3 May 2012), flowering (6 June 2012) and veraison (26 July 2012) (Figure 5) with sterile malt agar plug (control stems) or fungal strains after that a longitudinal wound (8 mm length, 1 mm deep) was performed with a sterile scalpel at level of the third internode. A 5 mm Ø malt agar or mycelial plug from the edge of a 5 days old actively growing fungal culture was then put into the wound and protected with parafilm. A total of 8 repetitions per treatment and one repetition per plant were performed. Observation of lesion development and reisolation tests (5 out of 8 repetitions) were performed on 16 October 2012 for C2, Ds and Np treatments from all phenological stages as described by Larignon and Dubos [59]. Samples for protein extraction (3 out of 8 repetitions), consisting in the inoculated internode (C2, Ds and Np) as well as of the correspondent non wounded internode of C1 stems were collected 20 days after the inoculation. Samples were frozen in the field with liquid nitrogen and subsequently stored at −80 °C. Before protein extraction, the amount of biological sample needed was ground to a fine powder in liquid nitrogen with a Mixer Mill MM 400 (Retsch, Haan, Germany).

**Table 4:** Description of sample codes.

Condition	Phenological Stage		
	G stage	Flowering	Veraison
Control 1	C1G	C1F	C1V
Control 2	C2G	C2F	C2V
*D.seriata* strain Bo98-1	DsG	DsF	DsV
*N.parvum* strain Np SV	NpG	NpF	NpV

Example: C1G—(non-wounded stem-G stage); C2G—(stem wounded but inoculated with sterile agar-G stage); DsG—(*D.seriata* strain Bo98-1-G stage); NpG—(*N.parvum* strain Np SV -G stage).

### 3.2. Two-Dimensional Gel Electrophoresis (2D) Analysis

#### 3.2.1. Protein Extraction

Total protein fraction of green stems and related two-Dimensional Gel Electrophoresis (2D) analysis were performed according to Magnin-Robert *et al.* [36].

#### 3.2.2. Image Analysis

Digitized images at 36.6 μm resolution were obtained using the GS-800 scanner and Quantity One 4.6.2 software (Bio-Rad, Hercules, CA, USA). Computerized 2D gel analysis, including spot detection and quantification, was performed using the PDQuest Basic 8.0.1 software (Bio-Rad, Hercules, CA, USA). The relative molecular mass was calibrated with internal protein markers (Precision Plus Protein Standards, Bio-Rad) after co-migration during the 2nd dimension. Quantification of detected protein spots was performed calculating the relative optical density × area (relative OD *×* area) in the gels. Normalization was set up according to the total spot density. Three different image analyses (one for each phenological stage) were performed. Since three biological repetitions per treatment (C1, C2, Ds and Np) were considered for the 2D approach, a total of 12 gel images per phenological stage were included in each analysis. Protein spots detected in at least 2 biological repetitions of a given treatment were considered for analysis and compared in all the treatments. Among the differentially expressed protein spots, 20 from the G stage, 15 from the flowering and 13 from the veraison (Figure 3) were subjected to in gel trypsin digestion followed by nanoLC−MS/MS analysis.

The mean relative OD × area ± SD (*n* = 3) values of each group were finally used to estimate relative expression level (relative OD × area %) of each protein spot among the groups. Differences among the means were evaluated by the Dunn’s Multiple Comparison Test after that the null hypothesis (equal means) was rejected in the Kruskal-Wallis test, assuming a significance of *p* ≤ 0.05. The relative expression ratio to the control (C1) in the other treatments was also estimated. Values ≥ |2| were discussed.

#### 3.2.3. Protein Identification by Mass Spectrometry

NanoLC-MS/MS analyses were performed on a nanoACQUITY Ultra-Performance-LC system (UPLC) coupled to a Q-TOF mass spectrometer (maXis, Bruker Daltonics, Bremen, Germany) mass spectrometer equipped with a nano electrospray source. The UPLC system was equipped with a Symmetry C18 precolumn (20 × 0.18 mm, 5 µm particle size, Waters, Milford, MA, USA) and an ACQUITY UPLC^®^ BEH130 C18 separation column (75 µm × 250 mm, 1.7 µm particle size, Waters). The solvent system consisted of 0.1% *v*/*v* formic acid in water (solvent A) and 0.1% *v*/*v* formic acid in acetonitrile (solvent B). Peptides were trapped during 3 min at 5 μL/min with 99% A and 1% B. Elution was performed at 60 °C at a flow rate of 450 nL/min, using a linear gradient of 6%–35% B over 9 min. The mass spectrometer was operating in positive mode, with the following settings: source temperature was set to 150 °C while drying gas flow was at 5 L/min. The nano-electrospray voltage was optimized to −4500 V. External mass calibration of the TOF was achieved before each set of analyses using Tuning Mix (Agilent Technologies, Palo Alto, CA, USA) in the mass range of 322–2722 *m*/*z*. Mass correction was achieved by recalibration of acquired spectra to the applied lock masses [methylstearate ([M + H]^+^ 299.2945 *m*/*z*) and hexakis (2,2,3,3,-tetrafluoropropoxy) phosphazine ([M + H]^+^ 922.0098 *m*/*z*)].

For tandem MS experiments, the system was operated with automatic switching between MS and MS/MS modes in the range of 100–2500 *m*/*z*. The two most abundant peptides (absolute intensity threshold of 2000), preferably doubly, triply and quadruply charged ions, were selected from each MS spectrum for further isolation and CID (Collision Induced Dissociation) fragmentation using nitrogen as collision gas. Ions were excluded after acquisition of two MS/MS spectra and the exclusion was released after 6 seconds. The complete system was fully controlled by Hystar 3.2 (Bruker Daltonics, Bremen, Germany).

Raw data collected during nanoLC-MS/MS analyses were processed, converted into mgf files with DataAnalysis (Bruker Daltonics, Bremen, Germany).

The MS/MS data were analyzed using the MASCOT 2.2.0. algorithm (Matrix Science, London, UK) and OMSSA (Open Mass Spectrometry Search Algorithm, National Institut of Health, Bethesda, MD, USA [60]) for search against an in-house generated protein database composed of protein sequences of *Vitis* and Fungi (taxonomy 3603 and 4751) and known contaminant proteins such as human keratins and trypsin, extracted from the NCBInr database (version 3; September 2013) and combined with reverse sequences for all entries using an in-house database generation toolbox available at https://msda.unistra.fr (total 3.409.536 entries).

Searches were performed without any molecular weight, or isoelectric point restrictions, trypsin was selected as enzyme, carbamidomethylation of cysteine (+57 Da) and oxidation of methionine (+16 Da) were set as variable modifications and mass tolerances on precursor and fragment ions of 10 ppm and 0.02 Da were used, respectively. Mascot and OMSSA results were loaded into the Scaffold software (version 2.2.0, Proteome Software Inc., Portland, OR, USA) and filtered in order to evaluate the false discovery rate [61]. Protein identification was confirmed when at least two peptides with high quality MS/MS spectra (less than 5 points below Mascot’s threshold score of identity at 95% confidence level, or an OMSSA *E*-value below −log(e5)) were identified. A more stringent filter was applied for single peptide identifications, the score of the unique peptide had to be higher than 10 points above Mascot’s threshold score of identity at 95% confidence level and an OMSSA *E*-value below −log(e10) was required. These thresholds led to protein identification with a false discovery rate of less than 1%.

#### 3.2.4. Functional Classification of Identified Proteins

A functional classification of the identified proteins was performed by using GenomeNet Database Resources (http:www.genome.jp/kegg) or according to their role described in the literature.

## 4. Conclusions

Involvement of the fungal pathogens (*N. parvum* and *D. seriata*) in the necrosis development was confirmed by their reisolation from the edge of the lesions. Results of the pathogenicity tests are in agreement with the proteome changes observed which also report a weakness status of the vine at the flowering stage. Indeed, a general trend of a down-accumulation of proteins (e.g., superoxide dismutase, SSP) was observed in green stems infected with *N. parvum* or *D. seriata*, especially at flowering, except for the over-accumulation of some HSP as well as a PRP 10 in DsF and some proteins involved in the primary metabolism in NpF. Inversely, strongest responses to the infection were particularly activated in G stage through an over-accumulation of primary metabolism proteins, defense and stress-related proteins (e.g., oxygen evolving enhancer, *S*-adenosylmethionine synthetase, 2-cys peroxiredoxin, pathogenesis related protein 10) in correlation with a lower development of the necrosis. According to the inoculated fungal strains, the disruption of the plant metabolism and the necrosis development are not closely related, indicating that different factors are involved in the virulence of the two fungal strains tested. Globally, the flowering stage seems to be the period of highest sensitivity to Botryosphaeria dieback agents possibly as consequence of the high metabolic activity oriented towards developing inflorescences. Little research has focused on the relationship between primary metabolism and defense responses, and therefore it would be interesting to unravel how primary metabolism occurring during the flowering stage influences defense responses.

## Supplementary Files

Supplementary File 1 Supplementary Table (XLSX, 387 KB)

## References

[1] Crous P.W., Slippers B., Wingfield M.J., Rheeder J., Marasas W.F.O., Philips A.J.L., Alves A., Burgess T., Barber P., Groenewald J.Z. (2006). Phylogenetic lineages in the Botryosphaeriaceae. Stud. Mycol..

[2] Slippers B., Wingfield M.J. (2007). Botryosphaeriaceae as endophytes and latent pathogens of woody plants: Diversity, ecology and impact. Fungal Biol. Rev..

[3] Phillips A.J.L., Alves A., Abdollahzadeh J., Slippers B., Wingfield M., Groenewald J.Z., Crous P.W. (2013). The Botryosphaeriaceae: Genera and species known from culture. Stud. Mycol..

[4] Úrbez-Torres J.R., Leavitt G.M., Voegel T.M., Gubler W.D. (2006). Identification and distribution of *Botryosphaeria* spp. associated with grapevine cankers in California. Plant Dis..

[5] Pitt W.M., Huang R., Steel C.C., Savocchia S. (2010). Identification, distribution and current taxonomy of Botryosphaeriaceae species associated with grapevine decline in New South Wales and South Australia. Aust. J. Grape Wine Res..

[6] Úrbez-Torres J.R. (2011). The status of Botryosphaeriaceae species infecting grapevines. Phytopathol. Mediterr..

[7] Pitt W.M., Huang R., Steel C.C., Savocchia S. (2013). Pathogenicity and epidemiology of Botryosphaerriaceae species isolated from grapevines in Australia. Aust. Plant Pathol..

[8] Rolshausen E., Akgül D.S., Perez R., Eskalen A., Gispert C. (2013). First report of wood canker caused by *Neoscytalidium dimidiatum* on grapevine in California. Plant Dis..

[9] Yan J.-Y., Xie Y., Zhang W., Wang Y., Liu J.-K., Hyde K.D., Seem R.C., Zhang G.-Z., Wang Z.-Y., Yao S.-W. (2013). Species of Botryosphaeriaceae involved in grapevine dieback in China. Fungal Divers..

[10] Larignon P., Fulchic R., Cere L., Dubos B. (2001). Observation on black dead arm in French vineyards. Phytopathol. Mediterr..

[11] Bertsch C., Ramirez-Suero M., Magnin-Robert M., Larignon P., Chong J., Abou-Mansour E., Spagnolo A., Clément C., Fontaine F. (2013). Grapevine trunk diseases: Complex and still poorly understood. Plant Pathol..

[12] Úrbez-Torres J.R., Leavitt G.M., Guerrero J.C., Guevara J., Gubler W.D. (2008). Identification and pathogenicity of *Lasiodiplodia theobromae* and *Diplodia seriata*, the causal agents of Bot canker disease of grapevines in Mexico. Plant Dis..

[13] Mohammadi H., Gramaje D., Banishashemi Z., Armengol J. (2013). Characterization of *Diplodia seriata* and *Neofusicoccum parvum* associated with grapevine decline in Iran. J. Agric. Sci. Technol..

[14] Epstein L.K., Sukhwinder K., Van der Gheynst J.S. (2008). Botryosphaeria-related dieback and control investigated in noncoastal California grapevines. Calif. Agric..

[15] Larignon P., Coarer M., Larbre C., Girardon K., Vigues V., Yobregat O. (2009). Identification sur le matériel végétal des sources d’inoculum des champignons associés aux maladies du bois. Phytoma.

[16] van Niekerk J.M., Calitz F.J., Halleen F., Fourie P.H. (2010). Temporal spore dispersal patterns of grapevine trunk pathogens in South Africa. Eur. J. Plant Pathol..

[17] Urbez-Torres J.R., Bruez E., Hutado J., Gubler W.W. (2010). Effect of temperature on conidial germination of Botryosphaeriaceae species infecting grapevines. Plant Dis..

[18] Larignon P., Dubos B. (2001). Le Black Dead Arm. Maladie nouvelle à ne pas confondre avec l’esca. Phytoma.

[19] Kuntzmann P., Villaume S., Larignon P., Bertsch C. (2010). Esca, BDA and Eutypiosis: Foliar symptoms, trunk lesions and fungi observed in diseased vinestocks in two vineyards in Alsace. Vitis.

[20] Úrbez-Torres J.R., Adams P., Kamas J., Gubler W.D. (2009). Identification, incidence, and pathogenicity of fungal species associated with grapevine dieback in Texas. Am. J. Enol. Vitic..

[21] Larignon P. Studies on the role of pruning wounds in infection by *Phaeocremonium aleophilum* and *Diplodia seriata* in France. Proceedings of the 8th International Workshop on Grapevine Trunk Diseases.

[22] Serra S., Mannoni M.A., Ligios V. (2008). Studies on the susceptibility of pruning wounds to infection by fungi involved in grapevine wood diseases in Italy. Phytopathol. Mediterr..

[23] Berger S., Sinha A.K., Roitsch T. (2007). Plant physiology meets phytopathology: Plant primary metabolism and plant-pathogen interactions. J. Exp. Bot..

[24] Lebon G., Wojnarowiez G., Holzapfel B., Fontaine F., Vaillant-Gaveau N., Clément C. (2008). Sugars and flowering in the grapevine (*Vitis vinifera* L.). J. Exp. Bot..

[25] Petit A.N., Baillieul F., Vaillant-Gaveau N., Jacquens L., Conreux A., Jeandet P., Clément C., Fontaine F. (2009). Low responsiveness of grapevine flowers and berries at fruit set to UV-C irradiation. J. Exp. Bot..

[26] Keller M., Viret O., Cole F.M. (2003). *Botrytis cinerea* infection in grape flowers: Defense reaction, latency, and disease expression. Phytopathology.

[27] Lebon G., Duchêne E., Brun O., Magné C., Clément C. (2004). Flower abscission and inflorescence carbohydrates in sensitive and non-sensitive cultivars of grapevine. Sex. Plant Reprod..

[28] Zappata C., Deléens E., Chaillou S., Magné C. (2004). Partitioning and mobilization of starch and N reserves in grapevine (*Vitis vinifera* L.). J. Plant Physiol..

[29] Riou C., Freyssinet G., Fevre M. (1991). Production of cell wall-degrading enzymes by the phytopathogenic fungus *Sclerotinia sclerotiorum*. Appl. Environ. Microbiol..

[30] Fasoli M., dal Santo S., Zenoni S., Tornielli G.B., Farina L., Zamboni A., Porceddu A., Venturini L., Bicego M., Murino V. (2012). The grapevine expression atlas reveals a deep transcriptome shift driving the entire plant into a maturation program. Plant Cell.

[31] Spagnolo A., Magnin-Robert M., Alayi T.D., Cilindre C., Schaeffer-Reiss C., van Dorsselaer A., Clément C., Larignon P., Suero-Ramirez M., Chong J. (2014). Differential responses of three grapevine cultivars to Botryosphaeria dieback. Phytopathology.

[32] Bolton M.D. (2009). Primary metabolism and plant defense-fuel for the fire. Mol. Plant Microbe Interact..

[33] Rojas C.M., Senthil-Kumar M., Vered T., Mysore K.S. (2014). Regulation of primary plant metabolism during plant-pathogen interactions and its contribution to plant defense. Front. Plant Sci..

[34] Forde B.B., Lea P.J. (2007). Glutamate in plants: Metabolism, regulation, and signalling. J. Exp. Bot..

[35] Spagnolo A., Magnin-Robert M., Alayi T.D., Cilindre C., Mercier L., Schaeffer-Reiss C., van Dorsselaer A., Clément C., Fontaine F. (2012). Physiological changes in green stems of *Vitis vinifera* L. cv. Chardonnay in response to esca proper and apoplexy revealed by proteomic and transcriptomic analyses. J. Proteome Res..

[36] Magnin-Robert M., Spagnolo A., Alayi T.D., Cilindre C., Mercier L., Schaeffer-Reiss C., van Dorsselaer A., Clément C., Fontaine F. (2014). Proteomic insights into changes in wood of *Vitis vinifera* L. in response to esca proper and apoplexy. Phytopathol. Mediterr..

[37] Roje S. (2006). *S*-Adenosyl-l-methionine: Beyond the universal methyl group donor. Phytochemistry.

[38] Tsunezuka H., Fujiwara M., Kawasaki T., Shimamoto K. (2005). Proteome analysis of programmed cell death and defense signaling using the rice lesion mimic mutant cdr2. Mol. Plant Microbe Interact..

[39] Figueiredo A., Fortes A.M., Ferreira S., Sebastiana M., Choi Y.H., Sousa L., Acioli-Santos B., Pessoa F., Verpoorte R., Pais M.S. (2008). Transcriptional and metabolic profiling of grape (*Vitis vinifera* L.) leaves unravel possible innate resistance against pathogenic fungi. J. Exp. Bot..

[40] Camps C., Kappel C., Lecomte P., Leon C., Gomes E., Coutos-Thevenot P., Delrot S. (2010). A transcriptomic study of grapevine (*Vitis vinifera* cv. Cabernet-Sauvignon) interaction with the vascular ascomycete fungus *Eutypa lata*. J. Exp. Bot..

[41] Margaria P., Abba S., Palmano S. (2013). Novel aspects of grapevine response to phytoplasma infection investigated by a proteomic and phospho-proteomic approach with data integration into functional networks. BMC Genomics.

[42] Robertson D., McCormick B.A., Bolwell G.P. (1995). Cell wall polysaccharide biosynthesis and related metabolism in elicitor-stressed cells of French bean (*Phaseolus vulgaris* L.). Biochem. J..

[43] Tenhaken R., Thulke O. (1996). Cloning of an enzyme that synthesizes a key nucleotide-sugar precursor of hemicellulose biosynthesis from soybean: UDP-glucose dehydrogenase. Plant Physiol..

[44] Reiter W.D., Vauzin G.F. (2001). Molecular genetics of nucleotide sugar interconversion pathways in plants. Plant Mol. Biol..

[45] Chávez Montes R.A., Ranocha P., Martinez Y., Minic Z., Jouanin L., Marquis M., Saulnier L., Fulton L.M., Cobbett C.S., Bitton F. (2008). Cell wall modifications in Arabidopsis plants with altered α-l-arabinofuranosidase activity. Plant Physiol..

[46] Gilbert L., Alhagdow M., Nunes-Nesi A., Quemener B., Guillon F., Bouchet B., Faurobert M., Gouble B., Page D., Garcia V. (2009). GDP-d-mannose 3,5-epimerase (GME) plays a key role at the intersection of ascorbate and non-cellulosic cell-wall biosynthesis in tomato. Plant J..

[47] Valtaud C., Foyer C.H., Fleurat-Lessard P., Bourbouloux A. (2009). Systemic effects on leaf glutathione metabolism and defence protein expression caused by esca infection in grapevines. Funct. Plant Biol..

[48] Letousey P., Baillieul F., Perrot G., Rabenoelina F., Boulay M., Vaillant-Gaveau N., Clément C., Fontaine F. (2010). Early events prior to visual symptoms in the apoplectic form of grapevine esca disease. Phytopathology.

[49] Magnin-Robert M., Letousey P., Spagnolo A., Rabenoelina F., Jacquens L., Mercier L., Clément C., Fontaine F. (2011). Leaf strip of esca induces alteration of photosynthesis and defence reactions in presymptomatic leaves. Funct. Plant Biol..

[50] Mittler R. (2002). Oxidative stress, antioxidants and stress tolerance. Trends Plant Sci..

[51] Lima M.R.M., Ferreres F., Dias A.C.P. (2012). Response of *Vitis vinifera* cell cultures to *Phaeomoniella chlamydospora*: Changes in phenolic production, oxidative state and expression of defence-related genes. Eur. J. Plant Pathol..

[52] Sauter M., Rzewuski G., Marwedel T., Lorbiecke R. (2002). The novel ethylene-regulated gene OsUsp1 from rice encodes a member of a plant protein family related to prokaryotic universal stress proteins. J. Exp. Bot..

[53] Mahomed W., van den Berg N. (2011). EST sequencing and gene expression profiling of defence-related genes from infected with *Phytophthora cinnamomi*. BMC Plant Biol..

[54] Roberts M.R., Salinas J., Collinge D.B. (2002). 14–3-3 proteins and the response to abiotic and biotic stress. Plant Mol. Biol..

[55] Wang W., Vinocur B., Shoseyov O., Altman A. (2004). Role of plant heat-shock proteins and molecular chaperones in the abiotic stress response. Trends Plant Sci..

[56] Duan Y.H., Guo J., Ding K., Wang S.-J., Zhang H., Dai X.-W., Chen Y.-Y., Govers F., Huang L.-L., Kang Z.-S. (2011). Characterization of a wheat HSP70 gene and its expression in response to stripe rust infection and abiotic stresses. Mol. Biol. Rep..

[57] Musetti R., di Toppi L.S., Ermacora P., Favali M.A. (2004). Recovery in apple trees infected with the apple proliferation phytoplasma: An ultrastructural and biochemical study. Phytopathology.

[58] Grenville-Briggs L.J., van West P. (2005). Biotrophic stages of oomycete–plant interactions. Adv. Appl. Microbiol..

[59] Larignon P., Dubos B. (1997). Fungi associated with esca disease in grapevine. Eur. J. Plant Pathol..

[60] Geer L.Y., Markey S.P., Kowalak J.A., Wagner L., Xu M., Maynard D.M., Yang X., Shi W., Bryant S.H. (2004). Open mass spectrometry search algorithm. J. Proteome Res..

[61] Elias J.E., Gygi S.P. (2007). Target-decoy search strategy for increased confidence in large-scale protein identifications by mass spectrometry. Nat. Methods.

